# Development and Maturation of Embryonic Cortical Neurons Grafted into the Damaged Adult Motor Cortex

**DOI:** 10.3389/fncir.2016.00055

**Published:** 2016-08-03

**Authors:** Nissrine Ballout, Isabelle Frappé, Sophie Péron, Mohamed Jaber, Kazem Zibara, Afsaneh Gaillard

**Affiliations:** ^1^Cellular Therapies in Brain Diseases Group, Experimental and Clinical Neurosciences Laboratory, Institut National de la Santé et de la Recherche Médicale, U1084Poitiers, France; ^2^Pole Biologie Sante, Université de Poitiers, U1084Poitiers, France; ^3^Faculty of Sciences, Lebanese UniversityBeirut, Lebanon; ^4^ER045 - Laboratory of Stem Cells, PRASE, DSSTBeirut, Lebanon; ^5^Centre Hospitalier Universitaire de PoitiersPoitiers, France

**Keywords:** motor cortex, cortical lesion, embryonic transplantation, maturation, proliferation, GFP

## Abstract

Injury to the human central nervous system can lead to devastating consequences due to its poor ability to self-repair. Neural transplantation aimed at replacing lost neurons and restore functional circuitry has proven to be a promising therapeutical avenue. We previously reported in adult rodent animal models with cortical lesions that grafted fetal cortical neurons could effectively re-establish specific patterns of projections and synapses. The current study was designed to provide a detailed characterization of the spatio-temporal *in vivo* development of fetal cortical transplanted cells within the lesioned adult motor cortex and their corresponding axonal projections. We show here that as early as 2 weeks after grafting, cortical neuroblasts transplanted into damaged adult motor cortex developed appropriate projections to cortical and subcortical targets. Grafted cells initially exhibited characteristics of immature neurons, which then differentiated into mature neurons with appropriate cortical phenotypes where most were glutamatergic and few were GABAergic. All cortical subtypes identified with the specific markers CTIP2, Cux1, FOXP2, and Tbr1 were generated after grafting as evidenced with BrdU co-labeling. The set of data provided here is of interest as it sets biological standards for future studies aimed at replacing fetal cells with embryonic stem cells as a source of cortical neurons.

## Introduction

The cerebral cortex is a six-layered structure composed of a large number of neurons classically divided into two major groups. In rodents, 70–80% of neurons are excitatory glutamatergic projection neurons and 15–20% are inhibitory GABAergic non-pyramidal interneurons (Hendry et al., 1987; Beaulieu, 1993). There is a correlation between the laminar position of cortical neurons and their connectivity (Jones, 1984; Marín and Rubenstein, 2003). As such, layer II/III callosal neurons project to the contralateral cortex, layer V neurons project to the striatum, midbrain pons, and spinal cord whereas layer VI neurons project to the thalamus (Greig et al., 2013). Projection cortical neurons from different layers express specific molecular markers (Molyneaux et al., 2007; Gaspard et al., 2008; Gaspard and Vanderhaeghen, 2011). For instance, Cux1 (cut-like homeobox 1) is a specific marker of projection neurons of the superficial layers II/III and IV (Leone et al., 2008), while Ctip2 is used as a marker of a subset of subcerebral projection neurons of deep layer V (Arlotta et al., 2005), Foxp2 is used as a marker of layer VI cortico-thalamic projection neurons (Ferland et al., 2003) and *Tbr1* is used as a marker of early-born neurons of the preplate and layer 6 (Bulfone et al., 1998). The complexity of cerebral cortex in terms of cell diversity and specificity of projection patterns is translated into difficulties to appropriately repair damaged pathways following injury or disease.

The cerebral cortex is the target of many neurological conditions such as trauma, stroke, and neurodegenerative disorders all associated with cell death and irreversible functional deficits. In response to cell loss, the capacity of axonal regrowth and spontaneous regeneration within the central nervous system (CNS) are limited (for review see Schwab, 2004). Neuronal transplantation appears as a promising therapeutic strategy to replace neurons and damaged pathways (Gaillard et al., 2004, 2007). The effectiveness of cortical transplantation depends on the capacity of grafted cells to develop into appropriate neurons expressing specific neurotransmitters and transcription factors and to reconnect damaged pathways. We have previously shown that transplantation of embryonic cortical neurons in the adult motor cortex immediately after injury allows the anatomical reconstruction of injured motor pathways and the development of efferent projections to appropriate cortical and subcortical host targets (Gaillard et al., 2007). While the full repertoire of projections by embryonic cortical grafted neurons appears to be produced after 6 weeks (Gaillard et al., 2007), no precise information about the dynamics of maturation and axonal projections development of the transplanted neurons is currently available. This information should provide a much-needed control reference regarding the appropriate development of cortical neurons derived from stem cells.

In this study, we aimed to characterize the spatio-temporal maturation of the different cell populations constituting the graft and their axonal outgrowth. For this, we performed lesions of the adult mouse motor cortex followed by cell transplantation of embryonic motor cortical tissues and undertook a time-course analysis 2, 4, 7, 14, and 30 days following transplantation. We performed BrdU labeling experiments in combination with the labeling of specific cortical layer identity markers to determine the temporal and phenotypical outcome of grafted cells. The progressive axonal outgrowth of grafted neurons up to 30 days post-transplantation was also examined. We provide reference information on the gradual neuronal differentiation and maturation of grafted neurons through the characterization of expression of markers of immature neurons, glutamatergic, and GABAergic phenotypes and layers specific molecular markers of cortical and sub-cerebral projection neurons.

## Materials and methods

### Animals

Housing of animals and all animal experimental procedures were carried out in accordance with the guidelines of the French Agriculture and Forestry Ministry (decree 87849) and of the European Communities Council Directive (86/609/EEC) and the procedures were approved by COMETHEA Poitou-Charentes. All efforts were made to reduce the number of animals used and suffering.

### Transplantation

Adult (4–6 months) C57BL/6 mice (*n* = 34, R Janvier) were anesthetized with avertin (250 mg per kg of body weight) and the motor cortex was aspirated from ~0.5 to 2.5 mm rostral to the Bregma and from 0.5 to 2.5 mm lateral to the midline with the corpus callosum left intact according to the protocol routinely used in our laboratory (Roger and Ebrahimi-Gaillard, 1994; Gaillard et al., 1998, 2007). Motor cortical tissue was harvested from embryonic day 14 transgenic mice embryos overexpressing the enhanced green fluorescent protein (GFP) under the control of a chicken beta-actin promotor (C57BL/6-TgN(beta-act-EGFP) Osb strain (Okabe et al., 1997). Motor cortical tissue was deposited immediately into the host lesion cavity (Gaillard et al., 2007).

### BrdU injections

To assess cellular proliferation in the graft, transplanted mice were given a single intraperitoneal injection of bromo-deoxyuridine (BrdU, Sigma, 50 mg/kg, 0.1 M NaOH, NaCl 0.9%) 2 (*n* = 3) or 4 (*n* = 3) days after transplantation. Animals were sacrificed 4 h after BrdU injection.

### Tissue preparation

Two, 4, 7, 14, and 30 days after transplantation, animals received a lethal dose of avertin and were transcardially perfused with 150 ml of saline (0.9%), followed by 250 ml ice-cold paraformaldehyde (PFA, 4%) in 0.1 M phosphate buffer (PB, pH 7.4). Brains were removed and post-fixed for a further 4 h in 4% PFA. Brains were cut into a 40 μm coronal section in 6 series with a vibrating microtome (Microm HM650V, Thermo Scientific) and stored at −20°C in a cryoprotective solution (20% glucose, 40% ethylene glycol, 0.025% sodium azide, 0.05 M phosphate buffer, pH 7.4).

### Immunohistochemistry

Immunostainings were performed as previously described (Gaillard et al., 2007, 2009). Free-floating sections were rinsed in 0.05 M Tris-buffered saline (TBS, pH 7.6) and incubated in TBS solution containing 0.3% Triton X-100 and 5% donkey serum at room temperature (RT) for 90 min to block nonspecific binding sites. Primary antibodies, diluted in blocking solution, were applied overnight at 4°C. Primary antibodies and dilution factors were as follows: rabbit anti-glial fibrillary acidic protein (GFAP, 1:500, Dako) a marker of astrocytes (Sofroniew and Vinters, 2010); guinea pig anti-doublecortin (DCX, 1:100, Abcam), a microtubule-associated protein localized in somata and processes of migrating and differentiating neurons (Brown et al., 2003); mouse anti-polysialylated-neural cell adhesion molecule (PSA-NCAM, 1:1000, AbCys) expressed by neuronal progenitors and by differentiating neurons (Bonfanti, 2006); mouse anti-NeuN (1:500, Millipore), a marker of mature neurons (Mullen et al., 1992); mouse or rabbit anti-GFP (1:1000, Molecular Probes); rabbit anti-CUX1 (1:800, Santa Cruz), a marker of superficial cortical layers (Leone et al., 2008); rabbit or rat anti-CTIP2 (rabbit, 1:500, rat, 1:300, Abcam), a marker of deep cortical layers V neurons (Arlotta et al., 2005); rabbit anti-FOXP2 (1:500, Abcam), a marker of deep cortical layer VI neurons (Ferland et al., 2003); Chicken anti-Tbr1 (1:500, Millipore), a transcription factor gene of the T box family that is highly expressed in early-born neurons of the preplate and layer 6 (Bulfone et al., 1998); rabbit anti-γ-aminubutyric acid (GABA, 1:1000, Sigma) and rabbit anti-vesicular glutamate transporter 1 (V-GLUT1) (1:2000, Synaptic System) marquers of GABAergic and glutamatergic neurons, respectively (Molyneaux et al., 2007); mouse anti-CAM Kinase II alpha (CAM-K II,1:300, Invitrogen), a proteinstrongly expressed in large pyramidal neurons in layer V of rodent cortex (Ochiishi et al., 1994) and rat anti-bromo-deoxyuridine (BrdU,1:200, Serotec) which incorporates into the DNA of dividing cells during the S-phase of the cell cycle (Nowakowski et al., 1989). Following primary antibody incubation, sections were washed three times in TBS for 15 min each and then incubated for 1 h at RT with the appropriate secondary antibodies. Secondary antibodies generated in donkey and conjugated either with Alexa Fluor® (1:500; Invitrogen) or Dylight® (1:500; Jackson Immunoresearch) were used. In order to limit non-specific labeling, which can arise from secondary antibody detection, a zenon kit (Invitrogen) was used to directly reveal neuronal nuclei (NeuN) using a primary antibody conjugated with an Alexa Fluor 555 fluorophore. Finally, the sections were rinsed 3 times in TBS and cover slipped with a 10% solution of polyvinyl alcohol containing 2.5% 1,4-diazabicyclo-2,2,2-octane (PVA/DABCO, both from Sigma).

For BrdU staining, before incubation with the primary antibody, sections were pre-treated with 2N HCl, 0.5% Triton X-100 in PBS for 30 min at 37°C followed by incubation with Borax (pH 8.6) for 30 min at RT and blocking with 3% bovine serum albumin (Sigma), 0.3% Triton X-100 in PBS 0.1 M, pH 7.4. Sections were covered with DePeX (VWR) mounting media.

### Imaging

Immunofluorescence sections were examined using an Axio Imager.M2 (Carl Zeiss). Areas of interest were further analyzed and imaged with a confocal laser-scanning microscope FV1000 (Olympus, France). For double or triple-stained sections, sequential multiple channel fluorescence scanning was used to prevent cross-talk between channels.

### Determination of graft size

Graft volumes (*V*) were estimated at various times after transplantation (2, 4, 7, 14, and 30 days) by outlining graft areas on every sixth coronal section using fluorescent microscopy at low power magnification and an image analysis system (Mercator, Explora Nova, La Rochelle, France). Graft volumes (*V*) were calculated according to the formula for two truncated cones (*V* = *A1* + 2*A2* + 2*A3* +…*An*)/2 × *h*) with h as the distance between two measured areas considering a section thickness of 40 μm.

### Fibers and cell counting

For each animal and in each area, using a high-magnification objective (X40), fibers density was quantified in 1 out of the 6 series of sections at various times after transplantation (2, 4, 7, 14, and 30 days). For this individual fibers were counted up to 1000 fibers in each area. At 30 days post-transplantation, the percentage of transplanted neurons expressing cortical transcription factors (TF) or neurotransmitters was determined through quantification within the GFP+ graft area in every six sections of the overlap between TF/NeuN, GABA/NeuN/, or glutamate/NeuN, respectively (>1000 NeuN + cells counted/animal; *n* = 5).

## Results

### Morphological characteristics of the graft and projections

**Figure 1:**
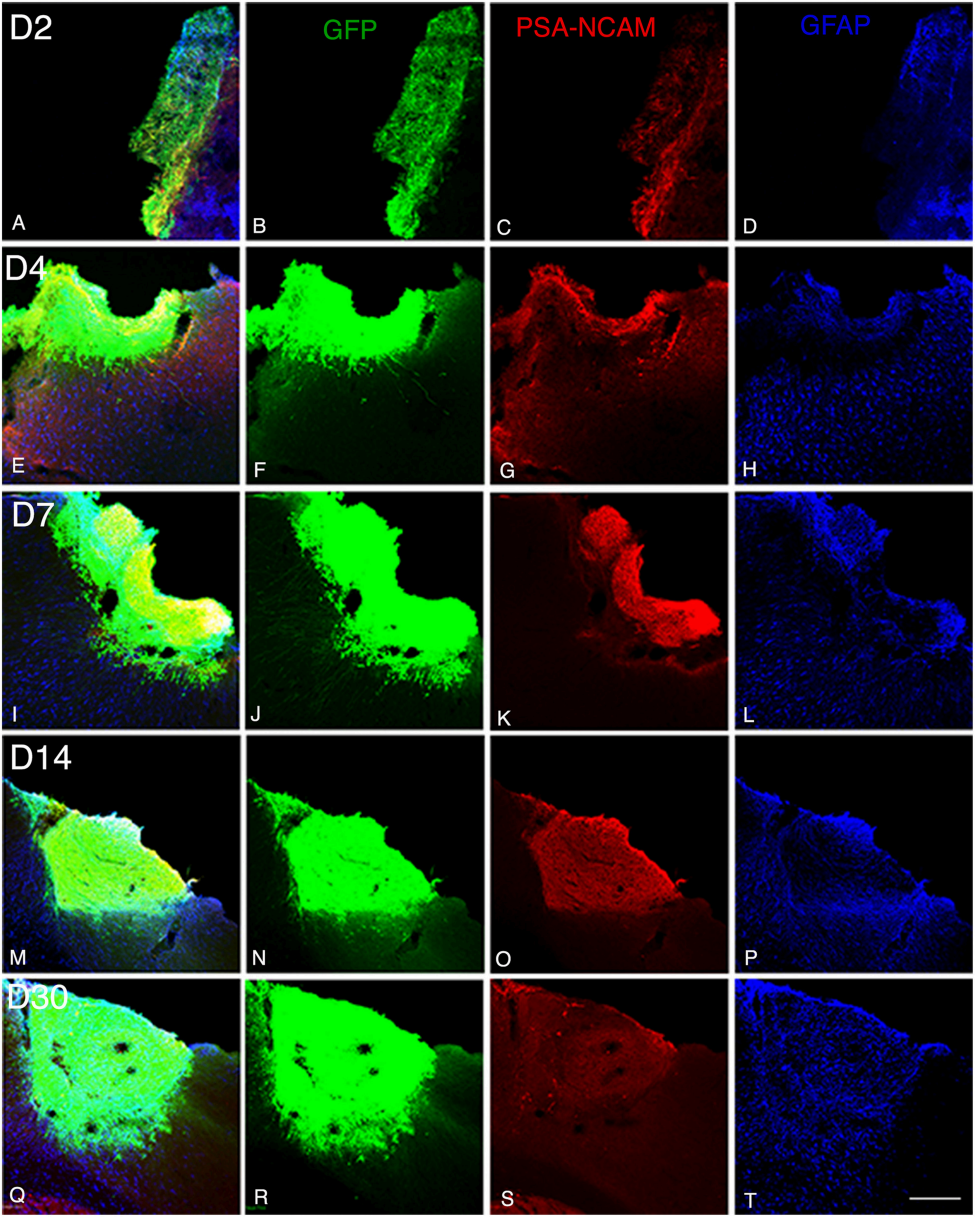
**Graft and graft-derived axonal projections at different time points following transplantation. (A–T)** Confocal images of GFP (green), PSA-NCAM (red), GFAP (blue) labeling from day 2 (D2) to day 30 (D30) after transplantation. **(A–P)** Grafts were composed of immature neural cell types as revealed by immunochemistry for GFP and PSA-NCAM. **(Q–S)** Grafted cells no longer expressed PSA-NCAM at day 30. **(A,D,E,H,I,L,M,P,Q,T)** Immunohistochemistry for the glial marker (GFAP) showed that the grafts also contained differentiated astrocytes. Note that not many GFAP expressing astrocytes were present at the host-graft interface. Scale bar: 130 μm.

GFP immunoreactivity was used to characterize graft and graft-derived axonal projections. At 2 days post-grafting, transplants appeared as a thin layer of GFP+ cells covering partially the base and/or the lateral walls of the cortical cavity (Figures 1A,B). The transplants were mainly located in the motor cortical areas I-II and the medial primary somatosensory cortical areas. The size of the grafts varied between 0.01 and 0.14 mm^3^ with an average volume of 0.07 ± 0.02 mm^3^. Three grafts out of a total of 5 showed short distance GFP+ projections in the adjacent motor and somatosensory cortices (Table 1).

**Table 1:** **Relative density of GFP+ fibers in cortical and subcortical brain regions receiving projections normally from the motor cortex**.

**Labeled structures**	**Days after transplantation**				
	**Day 2**	**Day 4**	**Day 7**	**Day 14**	**Day 30**
**CORTICAL PROJECTIONS**					
**Motor cortices**	*	*	****	****/**	****/*
**Somatosensory cortices**	*	*	***	****/**	****/*
**Cingulate cortex**			*	****/*	****/*
**Retrosplenial cortices**				**	**
**Orbital and Insular cortices**			*	**/*	**/*
**Prelimbic and infralimbic cortices**				****/*	****/*
**Auditory cortex**				**	***
**TELENCEPHALIC AND DIENCEPHALIC PROJECTIONS**					
**Corpus collasum**		*	*/*	****/**	****/**
**Caudate putamen**			*	****/**	****/*
**Internal capsule**				*	*
**Ventral lateral nucleus**				*	*
**Reticular thalamic nucleus**				*	*
**Cerebral peduncle**				*	*
**CORTICOSPINAL PROJECTIONS**					
**Spinal cord**					*

The number of asterisks indicates the fiber density within a given structure.

* , 1–50 fibers;

** , 50–100,

*** , 100–500 fibers;

**** , 500-1000 fibers.

*/, ipsilateral to the transplant; /*, contralateral to the transplant.

At 4 days post grafting, transplants were 2.9 times larger than those at 2 days post-grafting (average volume ± SEM: 0.2 ± 0.06 mm^3^; Figures 1E,F). Three out of five transplants filled the lower third of the cortical cavity, whereas the remaining two grafts appeared only as thin layers of GFP+ cells lying at the base or the lateral wall of the cavity. Compared to 2 days post-grafting, the number of GFP+ fibers located in the adjacent motor and somatosensory cortices was slightly increased (Table 1) and GFP+ fibers were found in these cortical areas in four out of five transplants. In fact, GFP+ fibers were found in the ipsilateral corpus callosum (CC) proceeding toward the midline in 2 cases whereas, few GFP+ fibers already reached the dorsal part of the caudate putamen (CPu) in one case. One transplant was very small in size (0.03 mm^3^) without any GFP+ fibers identified outside the transplant.

At 7 days post grafting, transplants were 1.6 times larger than those at 4 days post-grafting (average volume ± SEM: 0.33 ± 0.06 mm^3^; Figures 1I,J) and most of the transplants filled the whole cortical cavity. In addition, the density of GFP+ fibers was considerably increased in the motor and somatosensory cortices (Table 1). In four out of five cases, the number of fibers running into the CC was increased compared to day 4 post-transplantation. In two cases, GFP+ fibers crossed the midline and reached the contralateral CC. In three cases, increasing numbers of GFP+ fibers were identified in the dorsal CPu, and in one case rare fast developing fibers were also found in the ventrolateral part of the CPu.

At 14 days post grafting (Figures 1M,N), the graft size further increased by approximately two-fold (0.63 ± 0.08 mm^3^) in comparison to 7 days. Transplants filled the whole cortical cavity and the density of GFP fibers innervating the host was significantly increased (Table 1). Graft-derived GFP+ fibers were found in most of the brain areas normally innervated by the motor cortex, except distant areas such as the spinal cord. In seven out of eight cases, the number of fibers running into the contralateral CC was increased compared to day 7 post-transplantation. The number of GFP+ fibers was increased in the dorsal CPu in all cases (*n* = 8) and fibers also innervated the contralateral CPu in five animals. GFP+ fibers were also found in the ipsilateral and contralateral subventricular zone, in the internal capsule in the ventrolateral thalamic nucleus, in the cerebral peduncle and in the olfactory bulbs.

At 30 days post grafting (Figures 1Q,R), the graft size increased by ~1.6 times (1 ± 0.22 mm^3^) in comparison to 14 days. All cases (*n* = 5) showed far-reaching graft-derived GFP+ axonal growth, following specific paths (corpus callosum, internal and external capsule, cerebral peduncles) and reaching the specific cortical and subcortical targets that are normally innervated by neurons of motor cortex (Table 1).

### Development and maturation of the graft

We next focused on the temporal maturation of the grafted cells. For this, we labeled neuronal progenitors and differentiating neurons using antibodies directed against DCX and PSA-NCAM as well as mature neurons and astrocytes using antibodies directed against NeuN and GFAP, respectively.

**Figure 2:**
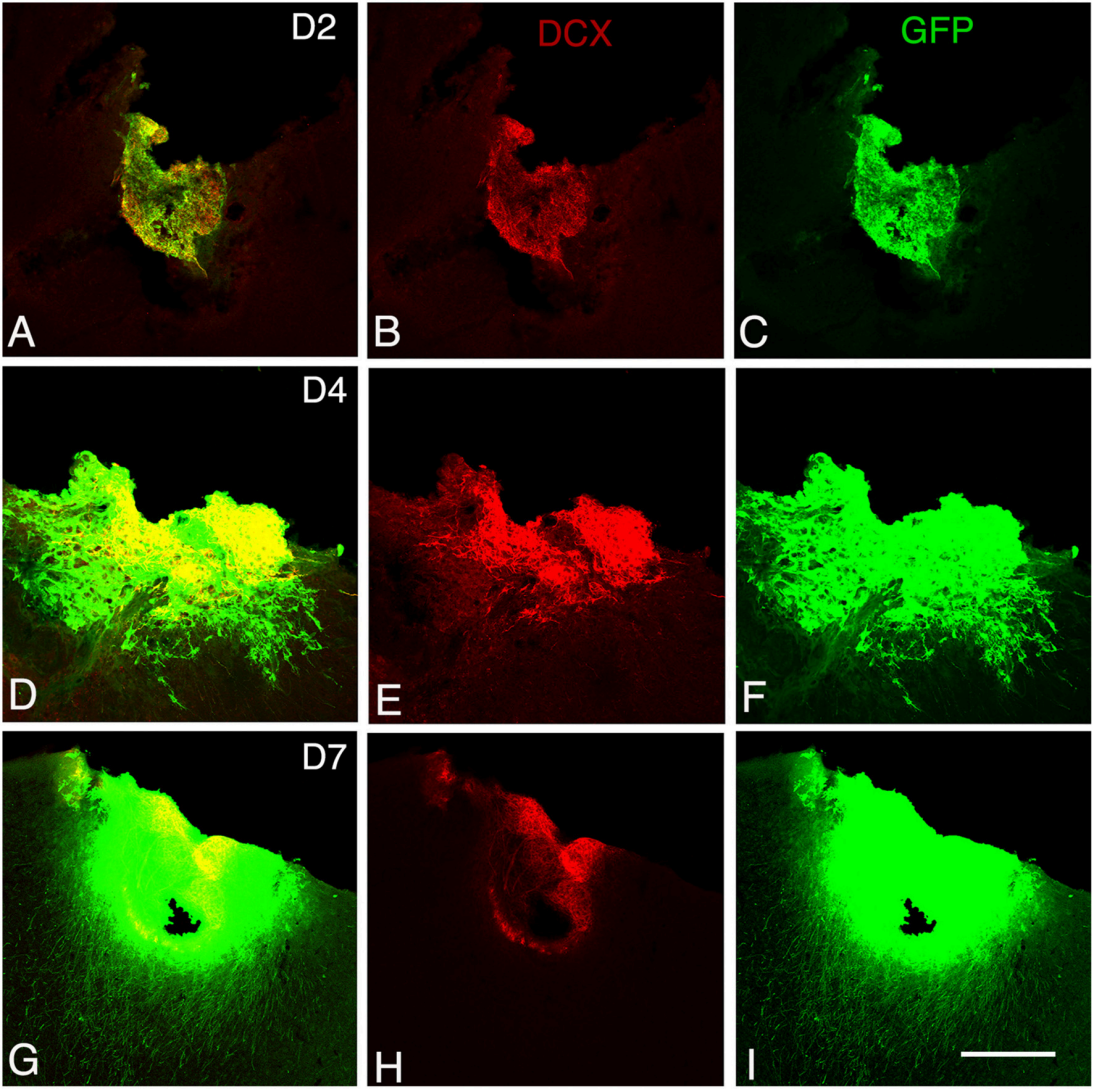
**Graft development at early stages**. Confocal images of GFP/doublecortin (DCX) labeling from 2 to 7 days (D2 to D7) after transplantation. **(A–I)** Many of the GFP (green) grafted cells with neuroblast morphology expressed DCX (red). Scale bars: 130 μm.

**Figure 3:**
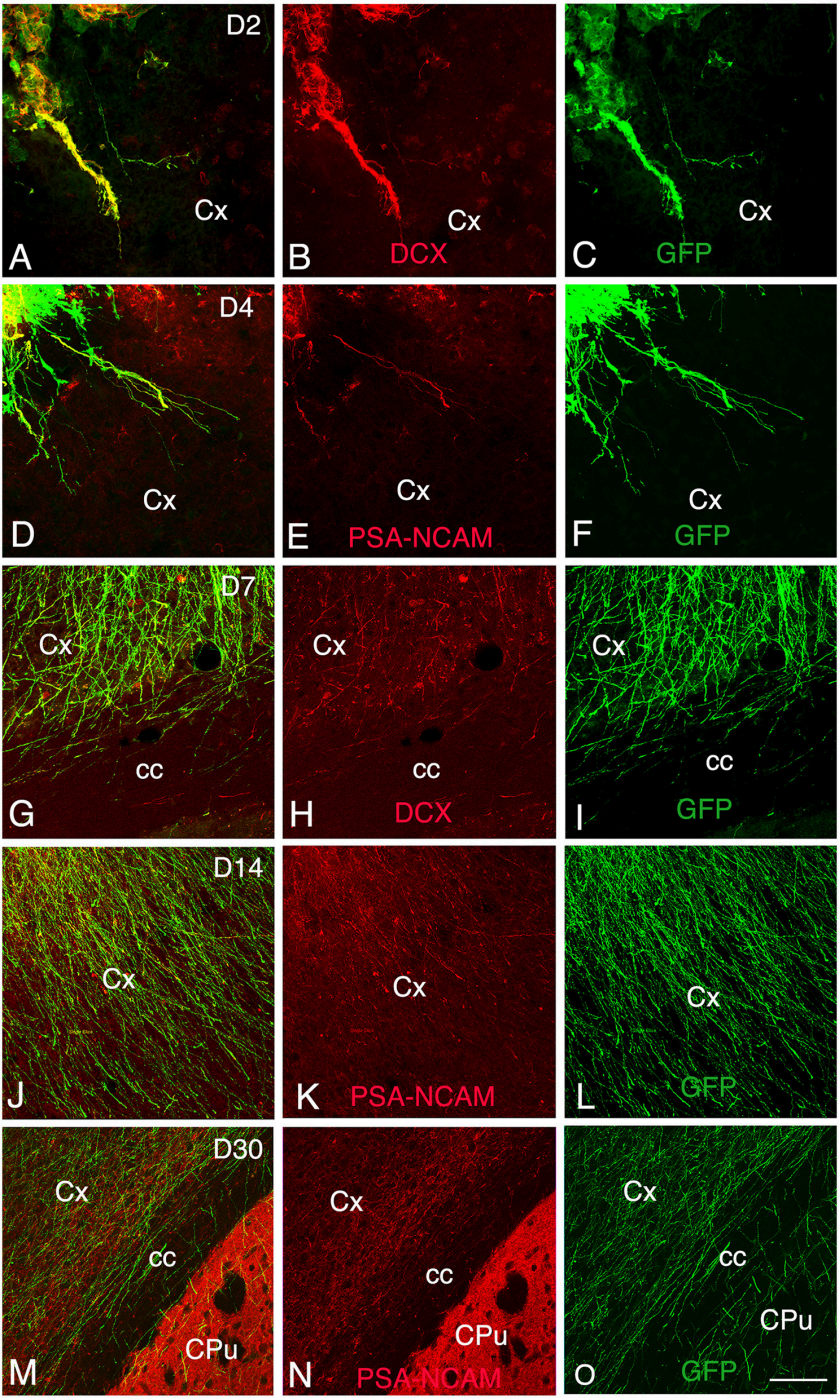
**Development and maturation of axons of grafted GFP neurons from day 2 (D2) to day 30 (D30) post-transplantation. (A–C, G–I)** Immunohistochemistry for GFP (green) and DCX (red) shows that many GFP+ fibers co-express DCX. (**D–F, J–O**) Immunohistochemistry for GFP (green) and PSA-NCAM (red) shows GFP axons co-expressing PSA-NCAM. GFP+ fibers co-expressing DCX or PSA-NCAM leave the graft and extend through the cortex and the corpus callosum ipsilateral to the transplant. Note that co-expression of DCX or PSA-NCAM by axons of grafted neurons decreased in relation with the post-transplantation time, indicative of the maturation of GFP axons. cc, corpus callosum; Cpu, caudate putamen; Cx, cortex; Scale bars: **A–C**, 55 μm; **D–O**, 80 μm.

At 2 days post-grafting, transplants appeared as densely packed GFP+ cell bodies. Many of the cells with neuroblast morphology in the transplant expressed both doublecortin (Figures 2A–C) and PSA-NCAM (Figures 1A,C). In all cases, a strong DCX expression was found in cell bodies and processes of the majority of the cells composing the transplants. In four out of five cases, PSA-NCAM expression appeared as a punctate membrane staining, mainly localized on the cell somata of most of the GFP+ cells. In all cases, sparse GFAP cells were found intermixed with neuroblasts within the whole transplants (Figures 1A,D). At this stage, the grafted cells were consistently negative for the mature neuronal marker NeuN. The first, but rare, GFP+ fibers growing out of the transplant co-expressed DCX (Figures 3A–C) but not PSA-NCAM.

At 4 days post-grafting, all transplants (*n* = 5) showed a large proportion of grafted cells expressing both DCX (Figures 2D–F) and PSA-NCAM (Figures 1E–G). Many grafted cells expressed GFAP whereas sparse host GFAP+ cells were found at the graft-host border (Figures 1E,H). At this time-point, many of the transplanted GFP neuronal axons highly expressed DCX and PSA-NCAM on their full-length processes (Figures 3D–F). None of the grafted GFP+ cells expressed the mature phenotype NeuN.

**Figure 4:**
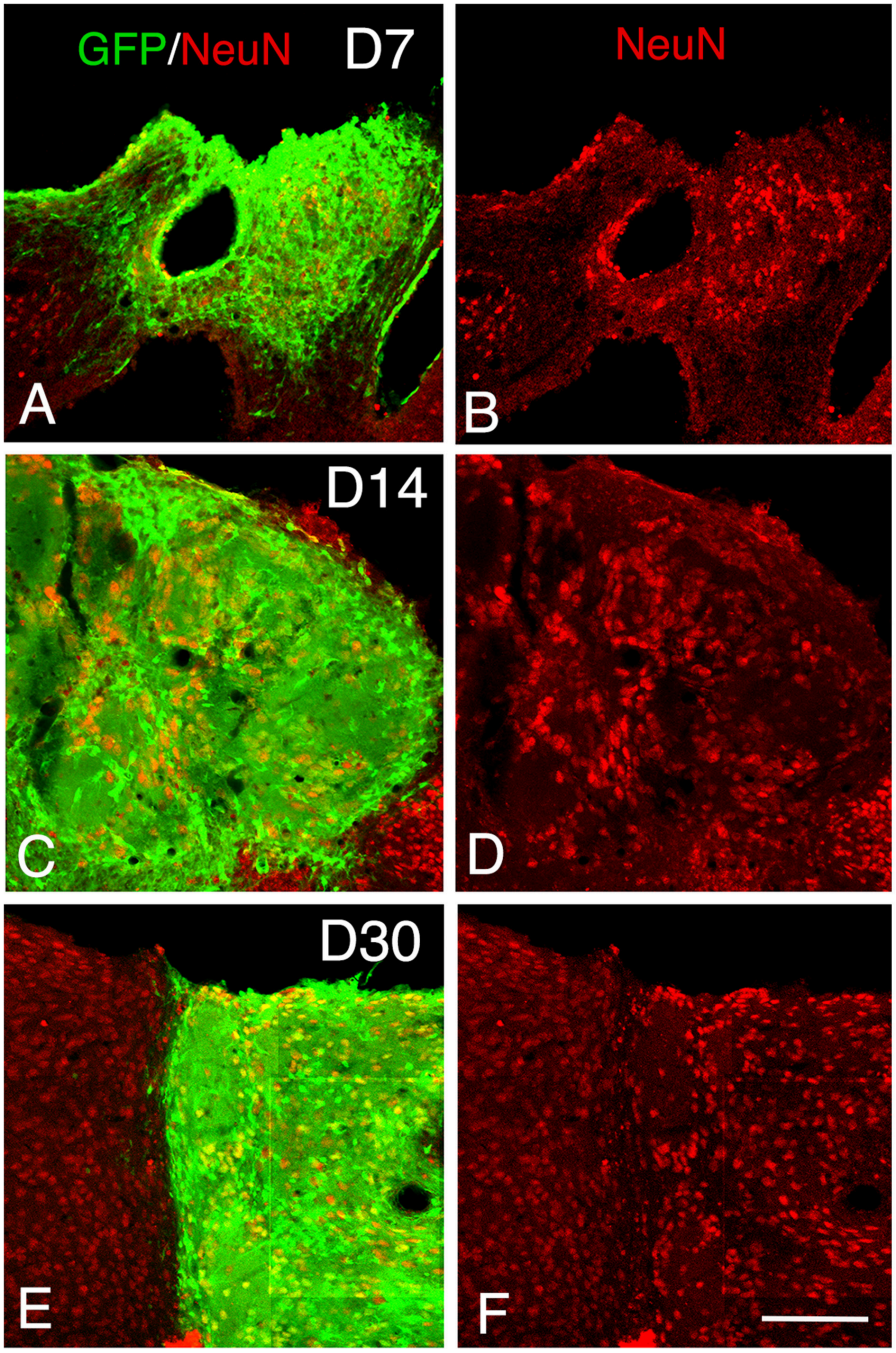
**Maturation of the transplanted neurons. (A–F)** Confocal images of GFP (green) and NeuN (red) from 7 to 30 days after transplantation showing an increase of the density of mature neurons within the graft in function of the time post-transplantation. Scale bar, 150 μm.

At 7 days post-grafting, grafted cells still strongly expressed both DCX (Figures 2G–I) and PSA-NCAM (Figures 1I,K). Mature neurons expressing NeuN were observed in two cases (Figures 4A,B). At this stage, the density of GFP+ fibers was considerably increased in the host adjacent cortex and many of those expressed both DCX (Figures 3G–I) and PSA-NCAM (Figures 3J–L). The level of GFAP expressing astrocytes in the graft was still sustained (Figures 1I,L) and astroglia appeared aligned at the periphery of the graft or within the septa separating PSA-NCAM highly stained lobules. Glial scar formation was never present at the host/transplant border (Figures 1I,L), 7 days after grafting.

At 14 days post-transplantation, the expression of DCX and PSA-NCAM was strongly decreased in transplants (Figures 1M,O). The vast majority of the grafted cells highly expressed NeuN (Figures 4C,D) and many grafted cells expressed GFAP (Figure 1P).

At 30 days post-transplantation, the grafts were almost exclusively populated by NeuN+ mature neurons (Figures 4E,F). At this time point, immature GFP+ neurons co-expressing DCX and PSA-NCAM were not detected in the transplants, indicating the full maturation of transplanted neurons 30 days after grafting (Figures 1Q–T, 3M–O).

### Cellular composition of the graft

**Figure 5:**
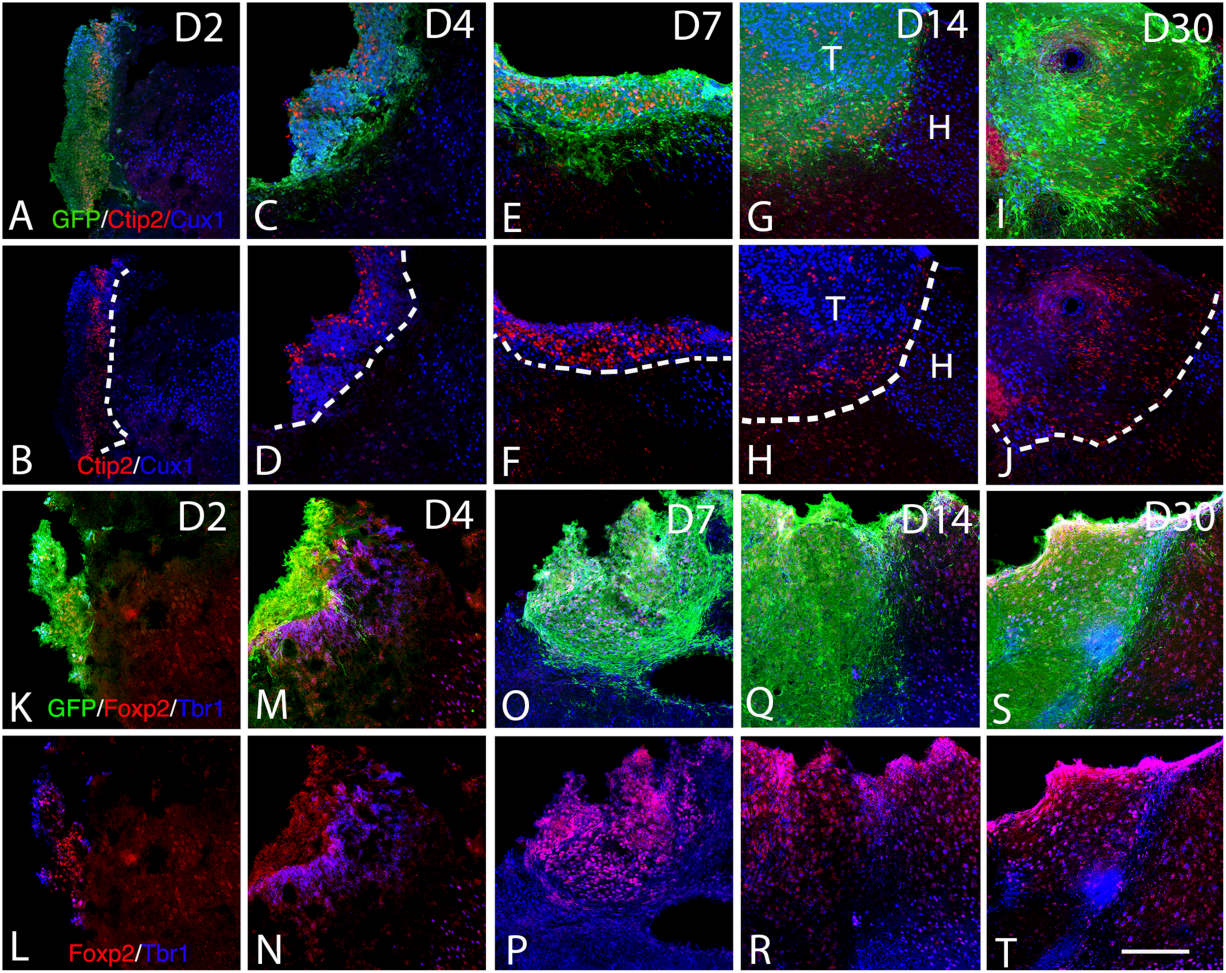
**Expression of cortical layer-specific transcription factors within the graft from D2 to D30 (A–R). (A–J)** Immunohistochemistry for GFP (green) and the transcription factors Ctip2 (red) and Cux1 (blue) within the graft and host cortex from D2 to D30. **(G,H)** The superficial and deep cortical layers of the host cortex are labeled by Cux1 (blue, layers II-IV) and Ctip2 (red, layers V-VI). **(G–J)**. The grafts were organized in clusters in which Ctip2 and Cux1 expression tend to be mutually exclusive. **(K–T)** Immunohistochemistry for GFP (green) and the transcription factors Foxp2 (red) and Tbr1 (blue) within the graft and host cortex from D2 to D30. **(K–T)** Tbr1+ and Foxp2+ cells were uniformly distributed through the grafts and the majority of the labeled cells co-expressed both markers corresponding to deep layer neurons (Tbr1, Foxp2). T, transplant; H, Host. Dashed lines indicate transplant-host border. Scale bar: 150 μm.

The adult cerebral cortex consists of six layers. Neurons from different layers are produced at different developmental time points. Earliest generated cortical neurons populate deep cortical layers whereas late born neurons generate the upper layers. Our results show that the grafted cells express the layer-specific cortical markers Ctip2 (Figures 5A–J), Foxp2 (Figures 5K–T), and CUX1 (Figures 5A–J) from the 2nd to the 30th-day post-transplantation. Despite the absence of laminar organization within the transplant, neurons expressing either Ctip2, Foxp2, or CUX1 were organized into distinct clusters within the transplant (Figures 5G–J), suggesting some level of organization. In some transplants, the expression of the transcription factors in the graft was in continuity with that of the host cortex. Indeed, Cux1+ cells within the graft were mostly located in the superficial part of the graft whereas CTIP2+ cells preferentially populated the deep part of the graft (Figures 5G,H).

**Figure 6:**
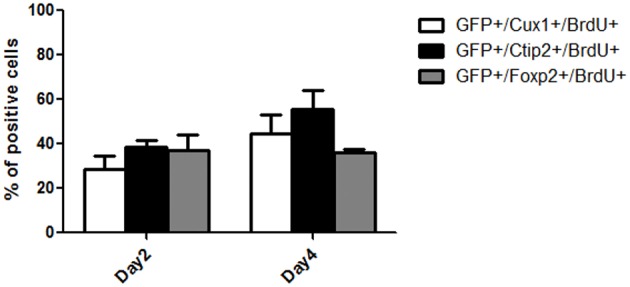
**Quantification of the percentage of BrdU+ cells within the grafts co-expressing Cux1, Ctip2, or Foxp2 at day 2 and 4 post-grafting**.

We next performed BrdU nuclear labeling experiments in the grafted mice to determine the date of birth of neurons with deep and superficial layers identity. At 2 days post grafting, a similar proportion of BrdU+ cells co-expressed CTIP2 (38.7 ± 2.3) or Foxp2 (37.1 ± 6.9) and a smaller population co-expressed Cux1 (28.3 ± 6) (Figure 6). At 4 days after grafting, the percentage of BrdU+ cells co-expressing CTIP2 increased compared to day 2 (55.3 ± 8.5), while the fraction of post-mitotic cells co-expressing Foxp2 remained unchanged (35.9 ± 1). Interestingly, the proportion of BrdU+ cells co-expressing the upper layer marker Cux1 tends to increase from day 2 to 4 post-grafting (44.7 ± 8.4; Figure 6), which is reminiscent of the delayed emergence of upper cortical layers during developmental corticogenesis. Together, this indicates a preserved diversity and differentiation potential of cortical progenitors following transplantation in the adult cortex.

**Figure 7:**
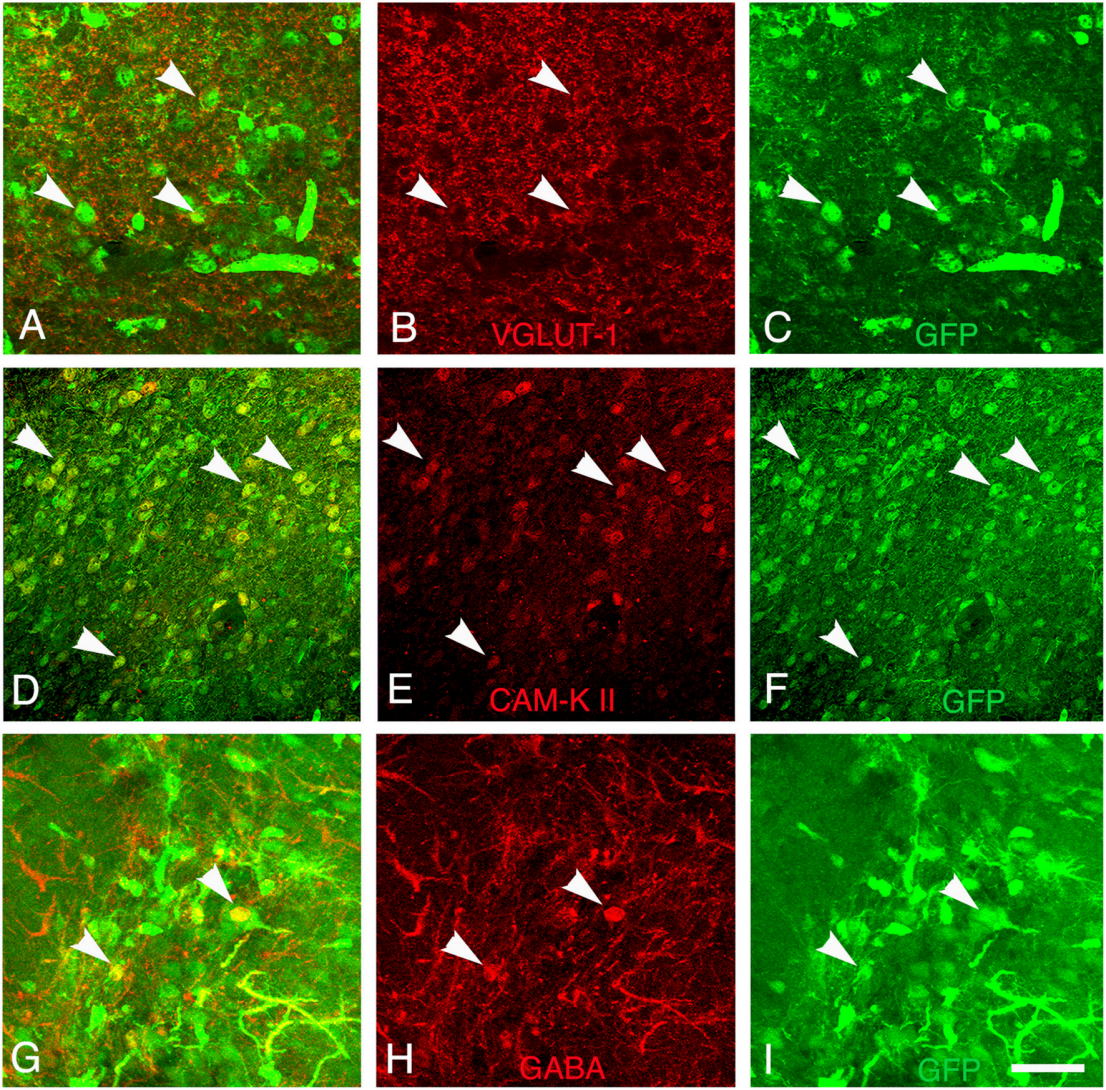
**Differentiation of grafted cells**. Identification of GFP+ cells (green) **(A–C)** co-expressing markers of glutamatergic neurons (V-GLUT1, red), **(D–F)** CAM-kinase II alpha, or **(G–I)** GABAergic neurons (GABA, red) at 30 days post-transplantation. Arrows show grafted neurons expressing V-GLUT-1, CAM Kinase II, or GABA. Scale bar: 40 μm.

Finally, the presence of both GABAergic interneurons and glutamatergic neurons within the grafts was evaluated by immunodetection of the neurotransmitter GABA and V-GLUT1, respectively. In addition, we analyze the cortical expression of CAM-kinase II alpha protein expressed in glutamatergic neurons and not in GABAergic neurons (Pinaudeau-Nasarre et al., 2002; Zou et al., 2002). This analysis was performed at the latest time-point (day 30 post-transplantation) to allow maturation of the grafted neurons. Results showed that the vast majority of grafted cells in the transplant were glutamatergic (Figures 7A–C) whereas only a few of them were GABAergic (Figures 7G–I). In addition to expressing mature neuronal markers, many grafted cells also expressed CAM-Kinase II alpha, characteristic of cortical projection neurons (Figures 7D–F). The quantification of the number of mature neuronal marker NeuN co-expressing V-GLUT-1 or GABA within the graft showed that 70% of the mature neurons in the transplant were glutamatergic whereas only 5% of NeuN+ neurons were GABAergic. The proportion of glutamatergic neurons within the graft matches the normal percentage of these neurons within the adult cortex.

## Discussion

In the past few years, the survival capacity of embryonic neurons transplanted in different regions of the adult brain has been demonstrated, and many studies have reported their repair potential at the neuroanatomical and functional levels (Lindvall et al., 1990; Plumet et al., 1993; Ebrahimi-Gaillard et al., 1995; Gaillard et al., 2007, 2009; Santos-Torres et al., 2009; Thompson et al., 2009; Jiménez-Díaz et al., 2011; Lu et al., 2012; Klein et al., 2013).

Here, we performed a time-course analysis, from 2 to 30 days following transplantation, in order to gain insight into the developmental course and maturation of cortical embryonic neurons after grafting into the damaged adult motor cortex.

We show here that as early as 2 weeks after grafting, cortical neuroblasts transplanted into damaged adult motor cortex developed specific projections to most of the cortical and subcortical targets. Transplanted embryonic cortical cells exhibited characteristics of immature neurons before differentiating into mature neurons with appropriate cortical phenotypes. Indeed, the grafted neurons expressed molecular markers that characterize neurons of different cortical layers and most of the mature neurons were glutamatergic while few were GABAergic.

At early time points, the presence within the graft of GFP+ cells that co-expressed either DCX or PSA-NCAM suggests ongoing neurogenesis as part of dynamic growth properties of the grafts. On the contrary, at 30 days post-grafting, the absence of GFP+ cells co-expressing DCX or PSA-NCAM is indicative of a complete state of maturation.

GFP+ cells along with cortical layer markers confirmed the presence of all cortical layers neurons within the grafts. The presence within the graft of BrdU+ cells co-expressing markers of cortical projection neuron identity, such as CTIP2, Cux1, FOXP2, and Tbr1, confirmed that all cortical subtypes were generated after grafting. Overall, this data indicates the generation of distinct and correct corticofugal neuron subtypes originating from the graft following transplantation in adult lesioned brain. The grafts also contained cells with mature neuronal and glial features as revealed by labeling with NeuN and GFAP. Importantly, the examination of neuronal phenotypes revealed a large population of glutamatergic neurons (70%) within the grafts as attested by the expression of VGLUT-1 or CAM-K II alpha. In the majority of cortical regions, alpha CAM-K II was expressed in the glutamatergic neurons but not in the GABAergic neurons. However, the density of GABAergic neurons within the cortical grafts was lower than in the intact cortex. This can be explained by the fact that the tissue for cortical transplantation was obtained from E14 embryos, a stage at which the majority of migrating GABAergic interneurons from the ventral telencephalon did not yet reach the cortical plate (Anderson et al., 1997; Marín and Rubenstein, 2003; Wonders and Anderson, 2006; Gelman et al., 2009). As such, there were few GABA precursors within the E14 transplant. This is of importance when one considers that the ratio between excitatory and inhibitory neurons in the cortex is critical to guarantee normal functioning of cortical circuitries. Indeed, impaired GABA-mediated neurotransmission has been implicated in many neurologic diseases, including epilepsy and intellectual disability (de Lanerolle et al., 1989; Spreafico et al., 1998). In this line, mouse GABAergic interneurons grafted into the brain of mice with temporal lobe epilepsy decreased seizure activity (Sebe and Baraban, 2011; Hunt et al., 2013; Tyson and Anderson, 2014).

For future development of cell replacement based therapies, there is a need for an unlimited on-demand source of transplantable cells that should be standardized and quality-tested prior to transplantation. Cortical neurons derived from embryonic stem cells (ESCs) and induced pluripotent stem cells (iPSCs) both offer great potential for cell therapy given their greater accessibility and standard use (Gaspard et al., 2008; Aboody et al., 2011; Espuny-Camacho et al., 2013; Michelsen et al., 2015). The success of stem cell-based neural repair strategies for neuronal replacement treatment following cortical damage will critically depend on the ability to generate not only specific cortical cell populations but also the maintenance of their correct ratios within the graft.

## Author contributions

NB, IF, and SP carried out the experiments and the analysis. AG designed and supervised the study, sought funding, and wrote the manuscript. KZ and MJ analyzed data and wrote the manuscript.

### Conflict of interest statement

The authors declare that the research was conducted in the absence of any commercial or financial relationships that could be construed as a potential conflict of interest.

## Abbreviations

CAM-K II: CAM Kinase II alpha
Cux1: cut-like homeobox 1
GFP: green fluorescent protein
GFAP: glial fibrillary acidic protein
DCX: doublecortin
PSA-NCAM: polysialylated form of the neural cell adhesion molecule
GABA: γ-aminubutyric acid
NeuN: neuronal nuclei
TBS: Tris-buffered saline
TF: transcription factors
V-GLUT1: vesicular glutamate transporter 1.
